# Clinical Use of Gastric Antisecretory Drugs in Hospitalized Pediatric Patients

**DOI:** 10.3390/jcm12010368

**Published:** 2023-01-03

**Authors:** Cristian Locci, Laura Cuzzolin, Gianluca Cheri, Laura Saderi, Giovanni Sotgiu, Roberto Antonucci

**Affiliations:** 1 Pediatric Clinic, Department of Medicine, Surgery and Pharmacy, University of Sassari, 07100 Sassari, Italy; 2 Department of Diagnostics & Public Health, Section of Pharmacology, University of Verona, 37134 Verona, Italy; 3 Clinical Epidemiology and Medical Statistics Unit, Department of Medicine, Surgery and Pharmacy, University of Sassari, 07100 Sassari, Italy

**Keywords:** antisecretory drugs, ranitidine, proton pump inhibitors, adverse drugs reactions, children

## Abstract

Antisecretory drugs are frequently used in the treatment of pediatric gastrointestinal disorders. This study was aimed to assess the prescribing patterns and the safety of ranitidine and proton pump inhibitors (PPIs) in a cohort of Italian pediatric patients. Children aged >1 month to <16 years that were admitted to our Pediatric Clinic between 2016 and 2018 were enrolled in this retrospective observational study. All data were obtained from medical records and a parent telephone questionnaire. The exclusion criteria included the use of antisecretory therapy at hospital admission, failure to collect the relevant clinical data, and failure to administer the questionnaire. This study included 461 subjects, who were divided into four age groups: <2 years, 2–5 years, 6–11 years, and ≥12 years. Ranitidine was prescribed in 396 (85.9%) patients, mainly for the acute treatment of gastrointestinal symptoms, and a PPI was given to 65 (14.1%) children to treat gastroesophageal reflux disease, gastritis/ulcer, or for gastroprotection. During the study period, the percentage of patients treated with ranitidine progressively increased, except in the 2–5-year age group. We observed eighty-seven adverse drug reactions (ADRs), 61 of which occurred in the ranitidine group and 26 in the PPI group. The most common ADR was constipation (*n* = 35), which occurred more frequently in children treated with PPIs and in the 6–11-year age group. Ranitidine was the most used antisecretory drug in all the age groups, especially for acute treatment. Conversely, PPIs were the drugs of choice for prolonged treatments. Further research should be focused on developing an effective and safer alternative to ranitidine.

## 1. Introduction

Gastric antisecretory drugs, mainly including histamine-2 receptor antagonists (H2RAs) and proton pump inhibitors (PPIs), are administered in pediatric patients with different gastrointestinal conditions (nausea, vomiting, abdominal pain, and hematemesis) [1], gastroesophageal reflux disease (GERD) [2], or in those at risk of gastric lesions caused by stress or pharmacological therapies [3].

H2RAs are less effective than PPIs for symptom relief, and may be used in older children and adolescents, although the occurrence of tachyphylaxis is a major drawback which seriously restricts their long-term use [4]. Moreover, these agents are associated with an increased risk of liver disease [5] and gynecomastia [6].

The available H2RAs, both prescribed and over-the-counter, are cimetidine, ranitidine, famotidine, and nizatidine, with the last two licensed for pediatric use only in the United States. In April 2020, the US Food and Drug Administration (FDA) requested the removal of all ranitidine products from the market because of a concern of higher than acceptable levels of a potential carcinogen, namely N-nitrosodimethylamine (NDMA) [7]. In Italy, ranitidine was licensed for use in infants and children aged >6 months (injectable solutions) or >3 years (oral administration) until September 2019, when all ranitidine preparations were withdrawn owing to the presence of NDMA [8]. On 17 September 2020, the Committee for Medicinal Products for Human Use (CHMP) of the European Medicine Agency (EMA) confirmed the suspension of ranitidine products in all European Union countries [9].

PPIs (omeprazole, esomeprazole, lansoprazole, pantoprazole, and rabeprazole) are among the most prescribed drugs worldwide [10]. Their use, approved for children aged >1 year, has increased during the last decade. They are the therapy of choice for pediatric GERD [11,12,13,14]. Moreover, PPIs do not cause tachyphylaxis [15], and might be appropriate for long-term therapy [16]. However, their increased prescription has raised safety concerns [17], including the risk of enteric and respiratory infections [18]. A review [19] showed that the adverse effects (e.g., headache, nausea, diarrhea, and constipation) occur in 34% of cases. In North America, the only PPI approved for use in children >1 year of age is esomeprazole [20]. In Italy, as well as in other European countries, the only drugs approved in children are omeprazole (>1 year of age) and esomeprazole (>12 years of age).

In order to assess the prescribing patterns and the safety of PPIs and H2RAs, a retrospective study was carried out in an Italian cohort of pediatric patients admitted to hospital. It is noteworthy that this study dates back to before ranitidine was withdrawn from the market.

## 2. Materials and Methods

### 2.1. Patients

Children aged from >1 month to <16 years that were admitted to the Pediatric Clinic at the Department of Medicine, Surgery and Pharmacy, University of Sassari, Italy, between 1 January 2016 to 31 December 2018, and exposed to PPI or H2RA treatment during the hospital stay, were enrolled in this retrospective observational study. Exclusion criteria included the use of antisecretory therapy at hospital admission, failure to collect the relevant clinical data, and the failed administration of the questionnaire.

Both the demographic and clinical data were collected from the medical records. Moreover, a questionnaire was administered to a parent of each participant after obtaining informed consent. The data were entered into an electronic database (Microsoft Excel, Microsoft Corporation, Redmond, WA, USA) for statistical analysis.

For each patient, the following variables were selected: sex, age at admission, body weight, indication for antisecretory therapy, drug regimen (route of administration, dosage and duration of therapy, and regimen changes), diagnostic tests, adverse drug reactions (ADRs), and post-discharge prescriptions.

In the case of a switch from PPIs to H2RAs and vice versa, the longer drug exposure was considered.

The study population was divided into the following age groups: <2 years, 2–5 years, 6–11 years, and ≥12 years. Furthermore, the participants were divided into two groups based on their drug exposure (i.e., H2RAs and PPIs).

For the purposes of this study, we used the definition of ADR proposed by Edwards and Aronson [21].

### 2.2. Diagnostic Tests

Diagnostic tests performed before or during therapy included esophagogastroduodenoscopy (EGDS), abdominal ultrasounds, plain abdominal radiograph, upper gastrointestinal series radiography (UGIS), and *Helicobacter pylori* stool antigen test.

### 2.3. Statistical Analysis

Qualitative variables were described with absolute and relative (percentage) frequencies, whereas for the quantitative ones the mean and the standard deviation were used for the variables with a normal distribution, and the median and interquartile range (IQR) were used for the variables with a non-normal distribution. To compare qualitative variables by age group, the Chi-squared (χ^2^) test or the Fisher’s exact test were used where appropriate. To compare the quantitative variables for multiple groups, the ANOVA test and the Kruskal–Wallis test were used for the parametric and non-parametric distributions, respectively. The Chi-squared (χ^2^) test or the Fisher’s exact test were used for the comparison of the qualitative variables by treatment group with ranitidine or PPI. The Student’s *t*-test and the Mann–Whitney test were used for the comparison of the quantitative variables for the parametric and non-parametric distributions, respectively. A two-tailed *p*-value < 0.05 was considered statistically significant. All the statistical analyses were carried out using STATA software version 17 (StataCorp LLC, College Station, TX, USA).

## 3. Results

During the study period, a total of 4645 patients were admitted and 559 (12%) of whom were eligible for the study. Ninety-eight patients were excluded for the following reasons: incomplete clinical data (*n* = 14); the use of antisecretory therapy at hospital admission (*n* = 32); the failed administration of the questionnaire (*n* = 52). Therefore, 461 patients were selected: the median (IQR) age was 7 (3–11) years, 57 subjects were aged <2 years, 138 were aged 2–5 years, 174 were aged 6–11 years, and 92 were aged ≥12 years. Two hundred and forty (52.1%) were males (Table 1). Ranitidine was prescribed to 396 (85.9%) patients, whereas PPIs (omeprazole, esomeprazole, pantoprazole, and lansoprazole) were prescribed to 65 (14.1%). The main indication for antisecretory drugs was gastrointestinal symptoms and signs, which were observed in more than half (*n* = 278; 60.3%), followed by gastroprotection for potentially gastrotoxic drugs (*n* = 100; 21.7%), and GERD (*n* = 16; 3.5%). The median (IQR) duration of the antisecretory therapy was 3 (1–6) days.

**Figure 1:**
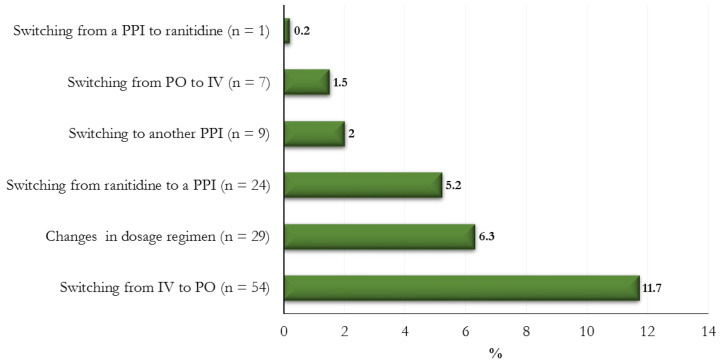
Changes in treatment prescriptions (%) during the study period.

Switching from intravenous (IV) to oral (PO) therapy (*n* = 54; 11.7%) was the most frequent regimen change, whereas ranitidine replaced PPIs in 24 (5.2%) patients (Figure 1).

Home therapy was prescribed in 79 of 461 (17.1%) patients, with 40 (51%) treated with ranitidine and 39 (49%) with a PPI, and was discontinued prematurely in only 5 (1.1%), 3 of whom were administered a PPI. Thirteen (2.8%) patients required a second treatment, 11 of whom received PPIs. The median (IQR) duration of home therapy was 15 (6–51) days.

During the study period, the number of subjects treated with antisecretory drugs increased from 111 in 2016 to 203 in 2018. Furthermore, the number of subjects treated with ranitidine doubled from 89 in 2016 to 169 in 2018, whereas the annual number of subjects treated with PPIs did not substantially change.

### 3.1. Comparison between the Different Age Groups

**Figure 2:**
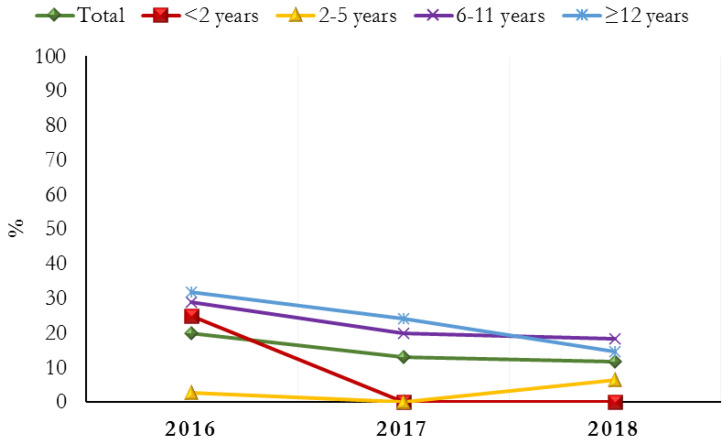
Subjects (%) treated with PPIs during the study period, stratified by age group.

**Figure 3:**
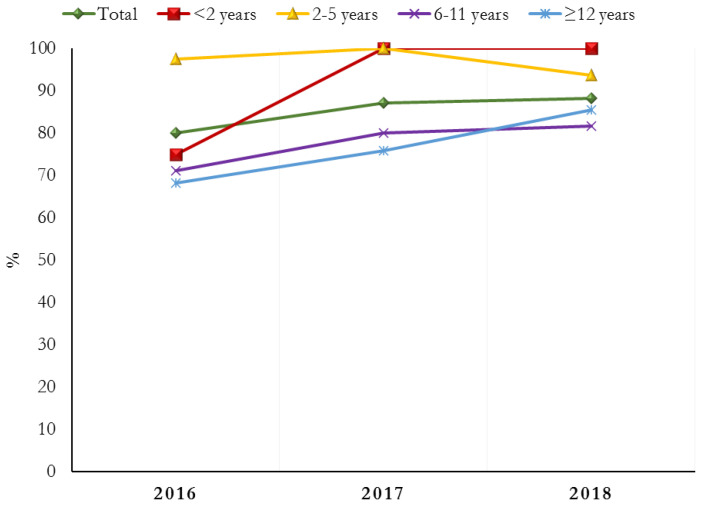
Subjects (%) treated with ranitidine during the study period, stratified by age group.

The percentage of subjects treated with PPIs progressively decreased in all age groups during the study period, with the only exception of the 2–5 years age group (Figure 2). Conversely, in the same time period, the prescription of ranitidine increased from 80.1% in 2016 to 88.2% in 2018, with the exception of the 2–5 years age group (Figure 3). Moreover, patients included in the 6–11 years and ≥12 years age groups were treated with significantly lower median doses of ranitidine IV (*p* = 0.0001) (Table 2).

With regards to the indications for the use of antisecretory drugs, the percentage of patients treated for gastrointestinal symptoms was significantly lower in children aged <2 years (*p* = 0.02). The percentage of patients who were exposed to antisecretory drugs for ingestion of caustics, irritants, and foreign bodies was significantly higher in children aged <2 years (*p* = 0.004).

Finally, the duration of antisecretory therapy was found to be significantly longer in children aged 6–11 years when compared with those aged 2–5 years (*p*= 0.02).

### 3.2. Comparison between the PPI Group and the Ranitidine Group

**Figure 4:**
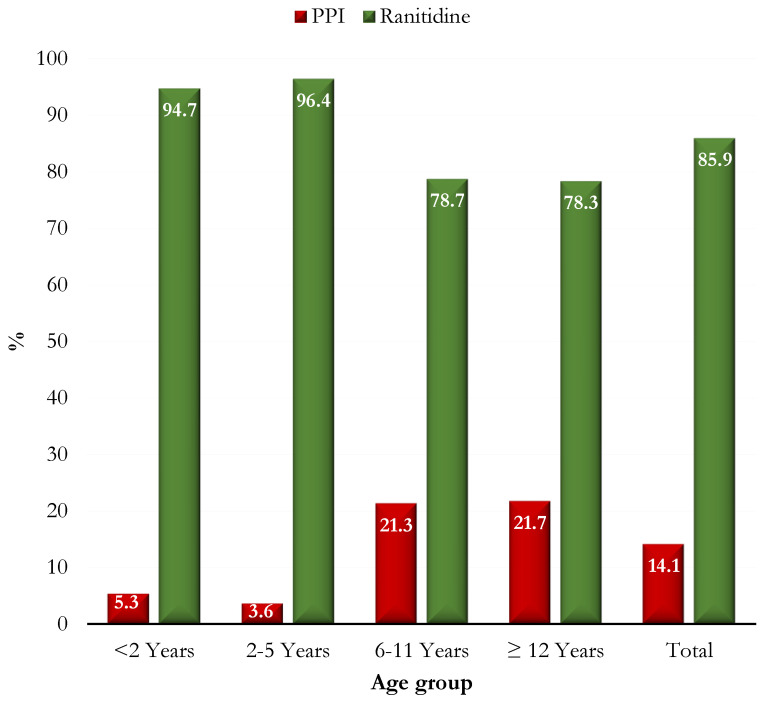
Percentage of patients on ranitidine or PPI therapy, stratified by age group.

Median age and weight were significantly higher in children in the PPI group (*p* < 0.0001) (Table 3, Figure 4). Furthermore, the median duration of treatment with PPIs was significantly greater than that of treatment with ranitidine (*p* < 0.0001).

The results of instrumental examinations (EGDS, abdominal ultrasound, bowel transit study, and abdominal X-ray) and laboratory tests (*Helicobacter pylori* stool antigen test) did not show significant differences between the two groups of patients.

Treatment was performed for gastrointestinal symptoms (nausea, vomiting, and abdominal pain) in a significantly higher percentage of subjects in the ranitidine group when compared to that in the PPI group (66.7% vs. 21.5%; *p* < 0.0001). Conversely, the percentage of patients treated for GER/GERD (1.8% vs. 13.9%; *p* < 0.0001), gastritis and peptic ulcer disease (0.8% vs. 7.7%; *p* = 0.002), gastroprotection (20% vs. 32.3%; *p* = 0.03), and other indications (5.3% vs. 15.4%; *p* = 0.003) was significantly higher in the PPI group.

Moreover, the percentage of subjects who continued therapy after discharge was significantly higher in the PPI group (60% vs. 10.1%; *p* < 0.0001). A significantly higher percentage of patients in the PPI group prematurely discontinued antisecretory therapy (4.6% vs. 0.5%; *p* = 0.02) and needed further treatment.

### 3.3. Adverse Reactions

A total of 87 (18.9%) ADRs were detected, including 61 (15.4%) in the ranitidine group and 26 (40.0%) in the PPI group (Table 4). The most incident ADR was constipation, which occurred in 35 (7.6%) cases, followed by diarrhea (*n* = 19; 4.1%), abdominal pain (*n* = 8; 1.7%), headache (*n* = 6; 1.3%), and vomiting (*n* = 6; 1.3%). Other ADRs (bradycardia, myalgia, skin rash, dizziness, neutropenia, nausea, asthenia, and pneumonia) were observed in 13 (2.8%) subjects. A significant difference between the two groups was observed only for constipation, which occurred more frequently in the PPI group (15.4% vs. 6.3%; *p* = 0.01).

Constipation occurred more frequently in children aged 6–11 years when compared to children aged 2–5 years (9.2% vs. 3.6%; *p* = 0.008). Diarrhea occurred less frequently in children aged 2–5 years, and was found to be more frequent in patients treated with PPIs, especially in the age group < 2 years.

Vomiting was significantly more frequent in the age group < 2 years than in the age group 2–5 years (5.3% vs. 0.0%; *p* = 0.006). It is noteworthy that, in the age group < 2 years, all children with vomiting were on therapy with ranitidine (Table 4).

## 4. Discussion

Prescription of gastric antisecretory drugs has progressively increased during the last decades [14,22,23], as well as their off-label use [24].

The present study was aimed at retrospectively assessing the use of PPIs and H2RAs in a large sample of hospitalized pediatric patients, during a time period when both ranitidine and PPIs were available. The ranitidine withdrawal has raised an important question: can PPIs be considered a suitable replacement for ranitidine in pediatric population? In fact, until now, clinical trials have not provided sufficient evidence of PPI efficacy and safety in younger children, especially for long-term outcomes [25,26].

Therefore, in order to better clarify these points, a special emphasis was given to prescription indication, dosage, treatment duration, variations in therapeutic regimen, adverse reactions, and post-discharge therapy.

First of all, in the three-year period considered in our study, we found an increasing trend in the prescription of ranitidine.

With regards to the indications for the use of antisecretory drugs, in contrast with the findings of Ruigómez et al. [27], we found that the percentage of patients treated with PPIs for gastrointestinal symptoms was significantly lower than that of the ranitidine group.

In the case of GERD, PPIs were more frequently prescribed than ranitidine (13.9% vs. 1.8%). This finding is consistent with the NASPGHAN-ESPGHAN guidelines published in 2018, which recommended PPIs for 4–8 weeks as a first-line treatment in the presence of typical GERD symptoms [28]. However, low quality evidence suggests that these drugs improve GERD symptoms in infants [29]. The study by Orenstein et al. [30] did not describe any differences in efficacy between lansoprazole and a placebo on the GERD symptoms in patients aged 1–12 months. In line with these findings, in our study, PPIs were rarely prescribed in children aged <2 years. In the age group < 12 years, the percentage of children treated with ranitidine was significantly higher than that of the children treated with PPIs, as in the study by Ruigómez et al. [27]. Moreover, we prescribed PPIs more frequently for “other indications”, including both eosinophilic esophagitis and post-operative complications of esophageal atresia, for which only PPIs are generally indicated [31,32,33].

In our patients, the dosage of ranitidine was within the range commonly reported in the literature for the oral route [19], but below the recommended range for the IV route (2–4 mg/kg/day) [34]. The prescribed dosage of PPIs was found to be within the range commonly used in pediatric practice [19].

Regarding the duration of treatment, our findings indicate that PPIs were preferentially administered for prolonged therapies. PPI therapy had a median duration of 16 days, whereas the median duration of ranitidine therapy was two days. This finding may be explained by the phenomenon of tachyphylaxis, which reduces the effectiveness of ranitidine in prolonged treatments [35,36]; conversely, PPIs do not exhibit this phenomenon [36]. In line with the study by Ruigómez et al. [27], we found that the percentage of patients treated with PPIs was higher than that of the ranitidine group when the indication was gastroprotection, gastritis, or peptic ulcer. In particular, PPIs were used with a significantly higher frequency in the treatment of peptic ulcer when compared to ranitidine (7.7% vs. 0.8%; *p* = 0.002). In the study by Litalien et al. [37], omeprazole was successfully prescribed in children and adolescents with gastric and duodenal ulcers refractory to H2RAs.

Among the variations in therapeutic regimens, switching from IV to PO therapy was the most frequent variation as the PO route was the one recommended at home. In the majority of patients, ranitidine was replaced by a PPI. This could be explained by the fact that PPIs are preferred over ranitidine in more prolonged therapies.

The percentage of subjects who continued home therapy as well as the median duration of the latter were significantly higher in the PPI group than in the ranitidine group. In longer home therapies, a compliance problem occurred: in particular, most patients who discontinued therapy and needed to repeat the course of treatment were found in the PPI group.

Regarding drug safety, patients exposed to PPIs showed more ADRs (40% vs. 34%) than those observed by Cohen et al. [19], whereas the frequency of ranitidine-related ADRs was lower in our cohort (15.4% vs. 23%).

The main ADR we recorded in both groups was constipation, followed by diarrhea and abdominal pain. In contrast, other authors reported that diarrhea, abdominal pain, nausea, headache, and vomiting were the most frequent ADRs [19].

The main study limitations are related to its retrospective design and the recall bias. Moreover, the present study was performed in a University Teaching Hospital equipped with a pediatric emergency department potentially resulting in a selection bias.

It should be noted that our study dates back to a period before ranitidine was withdrawn from the market. In fact, since 2019 the US FDA found NDMA levels exceeding the acceptable intake limit [38] in many batches of medications, including ranitidine. This led to widespread recalls of several products and to concerns among patients and clinicians, given that NDMA was classified as probably carcinogenic to humans. As a precaution, the FDA and the EMA requested manufacturers of ranitidine to remove all prescription and over-the-counter ranitidine products from the market [39,40], since NDMA concentrations appear to increase over time when stored above room temperature and could therefore result in dangerous levels [7].

Unfortunately, other H2Ras, such as cimetidine and famotidine are not authorized for use in pediatric patients, and therefore PPIs have become the only antisecretory drugs approved for use in children and adolescents in Italy as well as in other European countries. Our findings suggest a suboptimal role of PPIs in the treatment of pediatric acute conditions requiring rapid inhibition of gastric acid secretion.

## 5. Conclusions

Overall, the results of this study offer an insight into the clinical use of gastric antisecretory drugs in a pediatric hospital setting.

Ranitidine was the most frequently prescribed drug in all age groups, especially in children under 6 years of age, and it was found to be helpful for acute use due to its pharmacokinetic and pharmacodynamic properties [41]. On the other hand, PPIs were prescribed mainly to older children and adolescents, and in the case of prolonged therapy. In fact, PPIs do not exhibit the phenomenon of tachyphylaxis, they provide a more stable suppression of gastric acidity, and are characterized by a simpler dosage. However, PPIs showed a higher frequency of adverse events when compared to ranitidine. Moreover, doubts remain on PPI long-term effects [26], and their safety profile, particularly for chronic use, has yet to be adequately evaluated.

In conclusion, it seems reasonable to limit the empirical use of acid suppression therapy in children, particularly in infants. Further pharmacological research should be focused on the development of new fast-acting inhibitors of gastric acid secretion with proven efficacy and safety in children.

## Figures and Tables

**Table 1:** Demographic, clinical and treatment characteristics of the study population.

Demographic Characteristics		*n* = 461
Females, *n* (%)		221 (47.9)
Males, *n* (%)		240 (52.1)
Median (IQR) age, years		7 (3–11)
Age groups, *n* (%)	<2 year	57 (12.4)
	2–5 year	138 (29.9)
	6–11 year	174 (37.7)
	≥12 year	92 (20.0)
Median (IQR) weight, kg		24.2 (15.0–40.5)
**Treatments**		
Ranitidine, *n* (%)		396 (85.9)
Median (IQR) Ranitidine PO dose, mg/kg/day		4.5 (3.8–5.1)
Median (IQR) Ranitidine IV dose, mg/kg/day		1.5 (1.1–2.0)
PPI, *n* (%)		65 (14.10)
Median (IQR) PPI PO dose, mg/kg/day		0.6 (0.5–0.8)
Median (IQR) PPI IV dose, mg/kg/day		0.9 (0.8–1.0)
Median (IQR) PPI IV and PO doses, mg/kg/day		0.8 (0.5–1.0)
Median (IQR) variations therapy regimen		1 (1–1)
Median (IQR) duration of treatment, days		3 (1–6)
**Diagnostic Tests**		
Suggestive EGDS, *n* (%)		10 (58.8)
Suggestive abdominal ultrasound, *n* (%)		53 (89.8)
Suggestive Bowel Transit Study, *n* (%)		9 (60.0)
Suggestive abdominal X-ray, *n* (%)		7 (46.7)
*Helicobacter pylori* stool antigen test positive, *n* (%)		1 (6.3)
**Indication**		
Gastrointestinal symptom treatment, *n* (%)		278 (60.3)
Stress ulcer prevention, *n* (%)		13 (2.8)
Gastroprotection, *n* (%)		100 (21.7)
GER/GERD, *n* (%)		16 (3.5)
Caustics, irritants and foreign bodies ingestion, *n* (%)		15 (3.3)
Gastritis and peptic ulcer, *n* (%)		8 (1.7)
Other indications *, *n* (%)		31 (6.7)
**Home Therapy**		
Home therapy, *n* (%)		79 (17.1)
Ranitidine PO, *n* (%)		40 (51%)
Mean (SD) ranitidine PO, mg/kg/day		5.1 (1.5)
PPIs PO, *n* (%)		39 (49%)
Median (IQR) PPI dose, mg/kg/day		0.6 (0.4–0.9)
Median (IQR) duration of home therapy, days		15 (6–51)
Home therapy discontinued, *n* (%)		5 (1.1)
Repeated therapeutic courses, *n* (%)		13 (2.8)

PPI, Proton pump inhibitor. GER, Gastroesophageal reflux. GERD, Gastroesophageal Reflux Disease. EGDS, Esophagogastroduodenoscopy. IQR, Interquartile Range. * Other indications: adenomesenteritis, urticaria, angioedema, esophagitis, appendicitis, Meckel’s diverticulum, NSAID intoxication, mushroom poisoning, eosinophilic esophagitis, intestinal subocclusion, gastrointestinal complications in patients operated on for esophageal atresia, recurrent abdominal pain.

**Table 2:** Demographic, clinical and treatment characteristics of the study population stratified by age group.

	Age Groups				
	<2 Years (*n* = 57)	2–5 Years (*n* = 138)	6–11 Years (*n* = 174)	≥12 Years (*n* = 92)	*p*-Value
**Demographic Characteristics**					
Females, *n* (%)	25 (43.9)	56 (40.6)	89 (51.2)	51 (55.4)	0.10
Males, *n* (%)	32 (56.1)	82 (59.4)	85 (48.9)	41 (44.6)	
Median (IQR) age, years	1 (0–1)	4 (2–5)	9 (8–10)	14 (13–15)	-
Median (IQR) weight, kg	9.9 (7.9–11.3)	15 (13.3–17.6)	31.4 (25–37)	50 (46.0–57.5)	<0.0001 ^(1)^
**Treatments**					
Ranitidine, *n* (%)	54 (94.7)	133 (96.4)	137 (78.7)	72 (78.3)	<0.0001 ^(2)^
PPI, *n* (%)	3 (5.3)	5 (3.6)	37 (21.3)	20 (21.7)	
Median (IQR) Ranitidine PO dose, mg/kg/day	4.8 (4.2–6.1)	4.5 (4.0–5.4)	4.5 (3.8–5.0)	3.4 (2.6–4.3)	0.08
Median (IQR) Ranitidine IV dose, mg/kg/day	1.9 (1.6–2.4)	1.7 (1.3–2.4)	1.4 (1.1–1.8)	1.0 (0.9–1.2)	0.0001 ^(3)^
Esomeprazole, *n* (%)	1 (1.8)	4 (2.9)	7 (4.0)	1 (1.1)	0.65
Median (IQR) Esomeprazole PO dose, mg/kg/day	1.0 (−)	0.7 (0.6–1.2)	0.6 (0.3–0.6)	0.9 (−)	0.21
Omeprazole, *n* (%)	2 (3.5)	0 (0.0)	10 (5.8)	9 (10.0)	0.001 ^(4)^
Median (IQR) Omeprazole PO dose, mg/kg/day	0.8 (0.8–0.8)	-	0.6 (0.5–1.0)	0.4 (0.3–0.4)	0.003 ^(5)^
Pantoprazole, *n* (%)	0 (0.0)	0 (0.0)	18 (10.3)	10 (10.9)	<0.0001 ^(6)^
Mean (SD) Pantoprazole PO dose, mg/kg/day	-	-	0.6 (0.1)	0.7 (0.1)	0.17
Median (IQR) Pantoprazole IV dose, mg/kg/day	-	-	1.0 (0.8–1.1)	0.8 (0.5–0.9)	0.03
Lansoprazole, *n* (%)	0 (0.0)	1 (0.7)	2 (1.2)	0 (0.0)	0.86
Mean (SD) Lansoprazole PO dose, mg/kg/day	-	1.2 (−)	0.6 (0.3)	-	-
Median (IQR) PPI PO dose, mg/kg/day	0.8 (0.8–1.0)	0.8 (0.7–1.2)	0.6 (0.5–0.9)	0.4 (0.3–0.6)	0.01 ^(7)^
Median (IQR) PPI IV dose, mg/kg/day	-	1.0 (−)	1.0 (0.8–1.0)	0.8 (0.5–0.9)	0.08
Median (IQR) PPI IV and PO doses, mg/kg/day	0.8 (0.8–1.0)	0.9 (0.7–1.2)	0.8 (0.6–1.0)	0.5 (0.4–0.8)	0.01 ^(8)^
Median (IQR) variations therapy regimen	1 (1–1)	1 (1–1)	1 (1–1)	1 (1–1)	0.41
Drug dose changes, *n* (%)	1 (1.8)	7 (5.1)	14 (8.1)	7 (7.6)	0.33
From IV to PO, *n* (%)	7 (12.3)	15 (10.9)	18 (10.3)	14 (15.2)	0.68
From PO to IV, *n* (%)	0 (0.0)	2 (1.5)	4 (2.3)	1 (1.1)	0.79
From PPI to ranitidine, *n* (%)	0 (0.0)	0 (0.0)	1 (0.6)	0 (0.0)	1.0
From ranitidine to PPI, *n* (%)	2 (3.5)	3 (2.2)	13 (7.5)	6 (6.5)	0.16
From PPI to other PPI, *n* (%)	0 (0.0)	0 (0.0)	6 (3.5)	3 (3.3)	0.05
**Diagnostic Tests**					
Suggestive EGDS, *n* (%)	1 (50.0)	0 (0.0)	7 (63.6)	2 (66.7)	0.86
Suggestive abdominal ultrasound, *n* (%)	6 (100.0)	14 (87.5)	18 (81.8)	15 (100.0)	0.26
Suggestive Bowel Transit Study, *n* (%)	3 (75.0)	2 (66.7)	4 (50.0)	-	0.80
Suggestive abdominal X-ray, *n* (%)	0/3 (0.0)	4/4 (100.0)	3/6 (50.0)	0/2 (0.0)	0.02
*H. pylori* stool antigen test positive, *n* (%)	0 (0.0)	-	1 (8.3)	0 (0.0)	1.0
**Indication**					
Gastrointestinal symptom treatment, *n* (%)	25 (43.9)	93 (67.4)	103 (59.2)	57 (62.0)	0.02 ^(9)^
Stress ulcer prevention, *n* (%)	2 (3.5)	7 (5.1)	3 (1.7)	1 (1.1)	0.24
Gastroprotection, *n* (%)	12 (21.1)	28 (20.3)	41 (23.6)	19 (20.7)	0.90
GER/GERD, *n* (%)	5 (8.8)	2 (1.5)	6 (3.5)	3 (3.3)	0.11
Caustics, irritants and foreign bodies ingestion, *n* (%)	7 (12.3)	2 (1.5)	3 (1.7)	3 (3.3)	0.004 ^(10)^
Gastritis and peptic ulcer, *n* (%)	0 (0.0)	0 (0.0)	5 (2.9)	3 (3.3)	0.08
Other indications *, *n* (%)	6 (10.5)	6 (4.4)	13 (7.5)	6 (6.5)	0.44
**Adverse Reaction**					
Constipation, *n* (%)	2 (3.5)	5 (3.6)	16 (9.2)	12 (13.0)	0.03 ^(11)^
Diarrhea, *n* (%)	4 (7.0)	1 (0.7)	12 (6.9)	2 (2.2)	0.01 ^(12)^
Headache, *n* (%)	0 (0.0)	0 (0.0)	3 (1.7)	3 (3.3)	0.14
Abdominal pain, *n* (%)	0 (0.0)	4 (2.9)	2 (1.2)	2 (2.2)	0.55
Vomiting, *n* (%)	3(5.3)	0 (0.0)	2 (1.2)	1 (1.1)	0.04 ^(13)^
Other adverse reactions **, *n* (%)	3 (5.3)	4 (2.9)	1 (0.6)	5 (5.4)	0.04 ^(14)^
**Home Therapy**					
Home therapy, *n* (%)	11 (19.3)	20 (14.5)	30 (17.2)	18 (19.6)	0.72
Mean (SD) ranitidine PO, mg/kg/day	5.3 (1.3)	5.5 (1.5)	3.7 (1.5)	5.2 (1.4)	0.05
Median (IQR) PPI dose, mg/kg/day	1.0 (−)	1.0 (0.7–1.4)	0.6 (0.5–1.0)	0.4 (0.3–0.5)	0.008 ^(15)^
Median (IQR), duration of home therapy, days	10 (6–49)	7 (5.0–21.5)	35 (10–77)	17.5 (12–60)	0.02 ^(16)^
Home therapy discontinued, *n* (%)	0 (0.0)	2 (1.5)	0 (0.0)	3 (3.3)	0.06
Repeated therapeutic courses, *n* (%)	0 (0.0)	2 (1.5)	8 (4.6)	3 (3.3)	0.25
Median (IQR) duration of repeated therapeutic courses, days	2 (1–8)	1 (1–6)	3 (1–7)	3 (1.1–7.5)	0.04 ^(17)^
(1) Group < 2 years VS. Group 2–5 years *p*-value < 0.0001; Group < 2 years vs. Group 6–11 years *p*-value < 0.0001; Group < 2 years vs. Group ≥ 12 years *p*-value < 0.0001; Group 2–5 years VS. Group 6–11 years *p*-value < 0.0001; Group 2–5 years vs. Group ≥ 12 years *p*-value < 0.0001; Group 6–11 years vs. Group ≥ 12 years *p*-value < 0.0001; (2) Group < 2 years vs. Group 6–11 years *p*-value = 0.006; Group < 2 years vs. Group ≥ 12 years *p*-value = 0.007; Group 2–5 years vs. Group 6–11 years *p*-value < 0.0001; Group 2–5 years vs. Group ≥ 12 years *p*-value < 0.0001; (3) Group < 2 years vs. Group 6–11 years *p*-value = 0.0001; Group < 2 years vs. Group ≥ 12 years *p*-value < 0.0001; Group 2–5 years vs. Group 6–11 years *p*-value = 0.0001; Group 2–5 years vs. Group ≥ 12 years *p*-value < 0.0001; Group 6–11 years VS. Group ≥ 12 years *p*-value = 0.0005; (4) Group < 2 years vs. Group 2–5 years *p*-value = 0.03; Group 2–5 years vs. Group 6–11 years *p*-value = 0.004; Group 2–5 years vs. Group ≥ 12 years *p*-value = 0.0001; (5) Group 6–11 years vs. Group ≥ 12 years *p*-value = 0.002; (6) Group < 2 years vs. Group 6–11 years *p*-value = 0.01; Group < 2 years vs. Group ≥ 12 years *p*-value = 0.01; Group 2–5 years vs. Group 6–11 years *p*-value = 0.0001; Group 2–5 years vs. Group ≥ 12 years *p*-value = 0.001; (7) Group < 2 years vs. Group ≥ 12 years *p*-value = 0.04; Group 2–5 years vs. Group ≥ 12 years *p*-value = 0.02; (8) Group 2–5 years vs. Group ≥ 12 years *p*-value = 0.03; Group 6–11 years VS. Group ≥ 12 years *p*-value = 0.01; (9) Group < 2 years vs. Group 2–5 years *p*-value = 0.002; Group < 2 years vs. Group 6–11 years *p*-value = 0.04; Group < 2 years vs. Group ≥ 12 years *p*-value = 0.03; (10) Group < 2 years VS. Group 2–5 years *p*-value = 0.001; Group < 2 years vs. Group 6–11 years *p*-value = 0.0006; Group < 2 years vs. Group 2–5 years *p*-value = 0.002; Group < 2 years vs. Group ≥ 12 years *p*-value = 0.03; (11) Group 2–5 years vs. Group 6–11 years *p*-value = 0.008; (12) Group < 2 years vs. Group 2–5 years *p*-value = 0.01; Group 2–5 years vs. Group 6–11 years *p*-value = 0.006; (13) Group < 2 years vs. Group 2–5 years *p*-value = 0.006; (14) Group < 2 years vs. Group 6–11 years *p*-value = 0.02; Group 6–11 years vs. Group ≥ 12 years *p*-value = 0.01; (15) Group 2–5 years vs. Group ≥ 12 years *p*-value = 0.008; (16) Group 2–5 years vs. Group 6–11 years *p*-value = 0.007; Group 2–5 years vs. Group ≥ 12 years *p*-value = 0.04; (17) Group 2–5 years vs. Group 6–11 years *p*-value = 0.02.					

PPI, Proton pump inhibitor. GER, Gastroesophageal reflux. GERD, Gastroesophageal Reflux Disease. EGDS, Esophagogastroduodenoscopy. IQR, Interquartile Range. * Other indications: adenomesenteritis, urticaria, angioedema, esophagitis, appendicitis, Meckel’s diverticulum, NSAID intoxication, mushroom poisoning, eosinophilic esophagitis, intestinal subocclusion, gastrointestinal complications in patients operated on for esophageal atresia, recurrent abdominal pain. ** Other adverse reactions: bradycardia, myalgia, skin rash, dizziness, neutropenia, nausea, asthenia, pneumonia.

**Table 3:** Demographic clinical and treatment characteristics of the study population, stratified by treatment group.

	PPI (*n* = 65)	Ranitidine (*n* = 396)	*p* -Value
**Demographic Characteristics**			
Females, *n* (%)	26 (40.0)	195 (49.2)	0.17
Males, *n* (%)	39 (60.0)	201 (50.8)	
Median (IQR) age, years	10 (8–12)	6 (3–10)	<0.0001
Median (IQR) weight, kg	35.6 (22–45)	21.7 (14.3–37.6)	<0.0001
**Treatments**			
Median (IQR) variations therapy regimen	1 (1–2)	1 (1–1)	0.001
Drug dose changes, *n* (%)	4 (6.2)	25 (6.3)	1.0
From IV to PO, *n* (%)	6 (9.2)	48 (12.1)	0.50
From PO to IV, *n* (%)	2 (3.1)	5 (1.3)	0.26
From PPI to ranitidine, *n* (%)	1 (1.5)	0 (0.0)	0.14
From ranitidine to PPI, *n* (%)	22 (33.9)	2 (0.5)	<0.0001
From PPI to other PPI, *n* (%)	9 (13.9)	0 (0.0)	<0.0001
Median (IQR) total therapy duration, days	16 (5–559)	2 (1–5)	<0.0001
**Diagnostic Tests**			
Suggestive EGDS, *n* (%)	9 (69.2)	1 (25.0)	0.25
Suggestive abdominal ultrasound, *n* (%)	6 (75.0)	47 (92.2)	0.18
Suggestive Bowel Transit Study, *n* (%)	6 (54.6)	3 (75.0)	0.60
Suggestive abdominal X-ray, *n* (%)	1 (50.0)	6 (46.2)	1.0
*H. pylori* stool antigen test positive, *n* (%)	1 (9.1)	0 (0.0)	1.0
**Indication**			
Gastrointestinal symptom treatment, *n* (%)	14 (21.5)	264 (66.7)	<0.0001
Stress ulcer prevention, *n* (%)	4 (6.2)	9 (2.3)	0.10
Gastroprotection, *n* (%)	21 (32.3)	79 (20.0)	0.03
GER/GERD, *n* (%)	9 (13.9)	7 (1.8)	<0.0001
Caustics, irritants and foreign bodies ingestion, *n* (%)	2 (3.1)	13 (3.3)	1.0
Gastritis and peptic ulcer, *n* (%)	5 (7.7)	3 (0.8)	0.002
Other indications *, *n* (%)	10 (15.4)	21 (5.3)	0.003
**Home Therapy**			
Home therapy, *n* (%)	39 (60.0)	40 (10.1)	<0.0001
Median (IQR) duration of home therapy, days	40 (15–90)	9 (5–15)	<0.0001
Home therapy discontinued, *n* (%)	3 (4.6)	2 (0.5)	0.02
Repeated therapeutic courses, *n* (%)	11 (16.9)	2 (0.5)	<0.0001

* Other indications: adenomesenteritis, urticaria, angioedema, esophagitis, appendicitis, Meckel’s diverticulum, NSAID intoxication, mushroom poisoning, eosinophilic esophagitis, intestinal subocclusion, gastrointestinal complications in patients operated on for esophageal atresia, recurrent abdominal pain.

**Table 4:** Adverse drug reactions observed in the study population, stratified by age group and treatment group.

	Age Groups							
	<2 Years		2–5 Years		6–11 Years		≥12 Years	
Adverse Reaction	PPI (*n* = 3)	Ranitidine (*n* = 54)	PPI (*n* = 5)	Ranitidine (*n* = 133)	PPI (*n* = 37)	Ranitidine (*n* = 137)	PPI (*n* = 20)	Ranitidine (*n* = 72)
Constipation, *n* (%)	0 (0.0)	2 (3.7)	0 (0.0)	5 (3.8)	7 (18.9)	9 (6.7) **	3 (15.0)	9 (12.5)
Diarrhea, *n* (%)	1 (33.3)	3 (5.6)	0 (0.0)	1 (0.8)	4 (10.8)	8 (5.8)	0 (0.0)	2 (2.8)
Headache, *n* (%)	-	-	-	-	1 (2.7)	2 (1.5)	1 (5.0)	2 (2.8)
Abdominal pain, *n* (%)	-	-	1 (20.0)	3 (2.3)	1 (2.7)	1 (0.7)	1 (5.0)	1 (1.4)
Vomiting, *n* (%)	0 (0.0)	3 (5.6)	-	-	1 (2.7)	1 (0.7)	1 (5.0)	0 (0.0)
Other adverse reactions *, *n* (%)	-	3 (5.6)	-	4 (0.3)	-	1 (0.7)	3 (15.0)	2 (2.8)

PPI, Proton pump inhibitor. * Other adverse drug reactions: bradycardia, myalgia, skin rash, dizziness, neutropenia, nausea, asthenia, pneumonia. ** *p* = 0.02.

## Data Availability

The datasets generated and/or analyzed during the current study are available from the corresponding author on reasonable request.

## Abbreviations

PPIs	Proton pump inhibitors
ADRs	Adverse drugs reactions
H2RAs	Histamine-2 receptor antagonists
GERD	Gastroesophageal reflux disease
NDMA	N-nitrosodimethylamine
IQR	Interquartile range
